# Coincidental Coexistence of Human T-lymphotropic Virus Type 1 (HTLV-1)-Associated Myelopathy/Tropical Spastic Paraparesis and Myasthenia Gravis in a Patient With Chronic HTLV-1 Infection: A Case Report

**DOI:** 10.7759/cureus.88163

**Published:** 2025-07-17

**Authors:** Ignacio J Garma-Solis, Fanny S Zapata-Arceo, Victor M Ayuso-Diaz, Angelica Moreno-Enriquez

**Affiliations:** 1 Department of Internal Medicine, Institute for Social Security and Services for State Workers (ISSSTE) – Regional Hospital “Elvia Carrillo Puerto”, Mérida, MEX; 2 Research and Education Division, Medical Care and Research, Mérida, MEX; 3 Genomic-Metabolic Unit, Marista University of Mérida, Mérida, MEX

**Keywords:** antibody-mediated disease, htlv-1, human t-lymphotropic virus, myasthenia gravis, neuroinflammation, neurologic complications, neuromuscular autoimmune disease, rare case report, retroviral infection, tropical spastic paraparesis

## Abstract

Human T-lymphotropic virus type 1 (HTLV-1) infection profoundly alters central immune regulation via molecular mechanisms involving the viral proteins transactivator X and HTLV-1 basic leucine zipper factor, which promote the proliferation of autoreactive T lymphocytes and the dysfunction of regulatory T cells, resulting in persistent inflammation of the central nervous system. These alterations not only explain the occurrence of HTLV-1-associated myelopathy/tropical spastic paraparesis (HAM/TSP) but have also been associated with the development of autoimmune diseases such as myasthenia gravis (MG). While the connection between chronic HTLV-1 infection and MG is still anecdotal, a small number of case studies and limited molecular research suggest a potential link. Recent investigations have identified HTLV-1 *tax* and *pol* gene sequences in thymic tissue from MG patients, supporting the idea that the virus can persistently infect the thymus and interfere with the negative selection of T lymphocytes. Here, we present the case of a patient with HAM/TSP for over 12 years who subsequently experienced a myasthenic crisis, confirmed by the detection of anti-acetylcholine receptor autoantibodies. The patient responded favorably to treatment with acetylcholinesterase inhibitors. The absence of thymoma, together with a history of chronic retroviral infection, reinforced the potential role of HTLV-1 as a trigger for autoimmunity in the absence of structural abnormalities. This case illustrates the clinical and molecular convergence between retroviral infection and immune dysfunction, providing further support for a model of virally induced autoimmunity.

## Introduction

Human T-lymphotropic virus type 1 (HTLV-1) is an oncogenic retrovirus that is endemic to Asia, West Africa, Latin America, and the Caribbean. It establishes persistent infections by integrating its genome into the DNA of CD4+ T lymphocytes and, to a lesser extent, CD8+ T lymphocytes, and by expressing viral proteins such as the transactivator X (TAX) protein and the HTLV-1 basic leucine zipper factor (HBZ) protein. These proteins alter immune homeostasis by activating proinflammatory pathways, such as the nuclear factor kappa B (NF-κB) and signal transducer and activator of transcription 3 (STAT3) pathways, and by inhibiting apoptosis via B-cell lymphoma 2 (BCL2) and inducing abnormal clonal expansion of T lymphocytes [1,2]. Although evidence for NF-κB/STAT3 activation by TAX and HBZ in human thymic epithelial cells has been reported in vitro, further in vivo confirmation is required to fully elucidate their pathogenic roles [3].

HTLV-1 has an affinity for thymic tissue, where it directly infects thymic epithelial cells and modifies the central tolerance microenvironment. This results in the overexpression of cytokines and chemokines, such as interleukin 6 (IL-6), interleukin 15 (IL-15), and C-C motif chemokine ligand 4, which contribute to the disruption of the architecture of the thymus and interfere with the negative selection of autoreactive T lymphocytes [2,3].

In particular, HBZ inhibits the stable expression of forkhead box P3 (FOXP3) in regulatory T lymphocytes (Tregs) by repressing the transforming growth factor-beta (TGF-β)/SMAD signalling pathway, thereby favoring their conversion to a proinflammatory T-helper 1 (Th1) phenotype. While most of these findings originate from experimental and in vitro models, limited patient-based evidence indicates that comparable mechanisms may be present in vivo in individuals with HTLV-1-associated diseases, including HTLV-1-associated myelopathy/tropical spastic paraparesis (HAM/TSP) [2,4]. This Treg dysfunction plays a key role in the breakdown of immune tolerance and the development of autoimmunity.

One of the most distinctive neurological symptoms of chronic HTLV-1 infection is HAM/TSP. This chronic inflammatory disease involves progressive medullary axonal damage. It is associated with the migration of infected T lymphocytes to the central nervous system, guided by C-X-C motif chemokine ligand 10 (CXCL10) and driven by a microenvironment enriched in interferon-gamma (IFN-γ) and tumor necrosis factor-alpha (TNF-α). These factors perpetuate neurological damage [1,4].

By contrast, myasthenia gravis (MG) is an autoimmune disorder involving autoantibodies that target components of the postsynaptic membrane at the neuromuscular junction. These components include the acetylcholine receptor (AChR), muscle-specific kinase (MuSK), and low-density lipoprotein receptor-related protein 4 (LRP4). Under physiological conditions, autoreactive T lymphocytes are eliminated in the thymus by negative selection mechanisms [5,6]. However, MG patients with thymic hyperplasia or thymoma have a permissive environment for the formation of ectopic germinal centers, which promote the maturation of autoreactive B cells and subsequent autoantibody production [5-7].

Molecular studies have suggested the possible participation of HTLV-1 in the pathogenesis of MG. Truffault et al. [1] identified sequences of the viral *Tax* gene in the thymic tissue of 92% of MG patients and fragments of the *Pol* gene in over half of them using polymerase chain reaction and sequence analysis [6,7]. These findings support active HTLV-1 infection of the thymus as a central tolerance disruptor. Furthermore, functional studies have demonstrated that HTLV-1-infected epithelial cells can transfer the virus to CD4+ T lymphocytes via cell-to-cell contact, thereby amplifying aberrant immune activation [2].

From an immunopathological perspective, this connection is not coincidental. The combination of Treg dysfunction, viral persistence in the thymus, activation of autoreactive lymphocytes, and autoantibody formation suggests a model of virally induced autoimmunity in MG. Manca et al. previously suggested this hypothesis in 2002, when they warned of a possible retroviral involvement in the genesis of MG [3].

This article presents a case study of a patient with a 12-year history of tropical spastic paraparesis due to HTLV-1 infection. The patient experienced a myasthenic crisis, which was confirmed by the presence of anti-AChR autoantibodies, and responded well to treatment with acetylcholinesterase inhibitors. The case clearly demonstrates the link between chronic HTLV-1 infection and neuromuscular autoimmunity, which is supported by well-characterized molecular and cellular mechanisms.

## Case presentation

This is the case of a 60-year-old male patient with a documented 12-year history of HTLV-1-associated TSP. The diagnosis was confirmed in 2012 through the detection of anti-HTLV-1 antibodies in both blood and cerebrospinal fluid following the progressive onset of lower-limb stiffness, gait disturbance, and spasticity, initially accompanied by bladder urgency and mild sensory changes. There was no history of surgery, diabetes, hypertension, or other autoimmune conditions. The patient had received periodic botulinum toxin injections, most recently in March 2024, administered bilaterally to the lower limb adductors and gastrocnemius muscles (100 units total per session), with partial relief of spasticity.

Two weeks after this last injection, he developed progressive quadriparesis, predominantly affecting the lower limbs. Mild improvement was noted with rest, and no signs of intercurrent infection were reported. A transient adverse drug effect was initially suspected, and a wait-and-see approach was adopted. However, one week later, he developed right palpebral ptosis and episodic pharyngeal dysphagia, without noticeable diurnal variation.

Forty-eight hours before hospital admission, the patient experienced resting dyspnea accompanied by psychomotor agitation, prompting emergency department evaluation. He was treated with supplemental oxygen, which led to partial clinical improvement, and underwent diagnostic workup to rule out structural or metabolic causes.

On neurological examination, the patient exhibited dysarthria, right palpebral ptosis, and a positive curtain sign. No diplopia was observed, and the remainder of the cranial nerves were intact. There were no cerebellar signs. Muscle tone was globally normal; this was attributed to the effects of prior botulinum toxin treatment, as the patient had longstanding spasticity. Manual muscle strength testing revealed proximal > distal weakness, graded 4/5 in the upper limbs and 3/5 in the lower limbs. Osteotendinous reflexes were mildly diminished throughout, and both superficial and deep sensation remained preserved. No pathological reflexes were elicited.

To better document clinical severity, a Myasthenia Gravis Activities of Daily Living (MG-ADL) score of 8 was recorded on admission. Quantitative Myasthenia Gravis (QMG) scoring was not performed at that time. At discharge, the MG-ADL score improved to 3.

**Figure 1:**
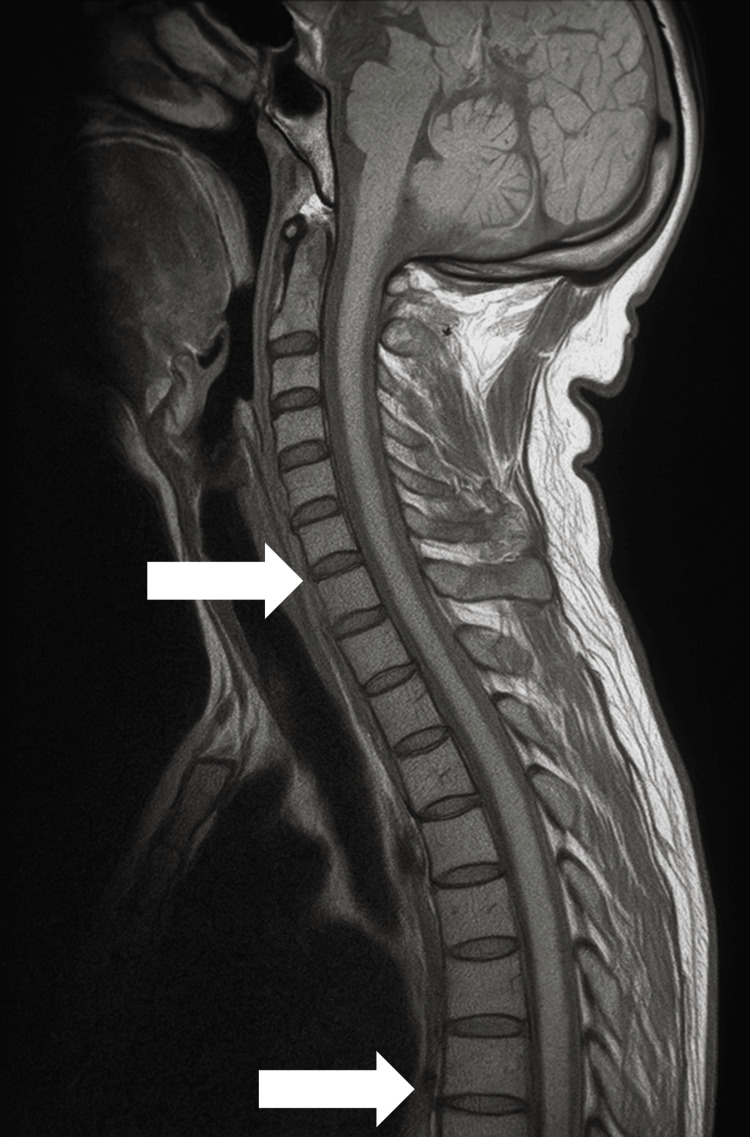
T1-weighted sagittal MRI scan of the cervical spine. Preserved cervical spinal cord with no compressive lesions, intramedullary signal alterations, or significant atrophy. The upper arrow indicates the mid-cervical cord, where spinal morphology and signal are preserved. The lower arrow shows an intervertebral space with no disc protrusion or canal stenosis. No overt signs of cervical myelopathy are visible on this sequence. Thoracic spine imaging was not available for evaluation of spinal cord atrophy often associated with human T-lymphotropic virus type 1-associated myelopathy/tropical spastic paraparesis.

A brain and cervical spine MRI (T1-weighted sequence) revealed no compressive lesions or demyelinating changes. Unfortunately, T2-weighted or short tau inversion recovery sequences were not available due to the study being performed at a private external center. Figure 1 displays the available sagittal T1 image, confirming preserved vertebral alignment and spinal cord continuity. There were no signs of cervical spinal cord atrophy or myelopathy in the available field of view. However, thoracic spine sequences were not obtained, and subtle signs of thoracic cord thinning cannot be definitively excluded. A chest CT was not performed; therefore, thymic morphology could not be assessed.

Given the patient’s clinical evolution, a diagnosis of MG was proposed. Although the repetitive nerve stimulation test did not show a significant decrement, the immunological panel confirmed the presence of anti-AChR antibodies, with a serum concentration of 3.2 nmol/L. The assay used was a radioimmunoprecipitation test, with a laboratory cut-off of ≥0.5 nmol/L for positivity. This result confirmed the diagnosis of seropositive generalized MG. Relevant laboratory and respiratory function test results are summarized in Table 1.

**Table 1:** Relevant laboratory findings and functional tests in a patient with a newly diagnosed case of myasthenia gravis. The key findings of the diagnostic approach are shown, including the specific serology test for anti-acetylcholine receptor (AChR) antibodies, respiratory function tests, and repetitive stimulation electromyography. Anti-AChR positivity was confirmed using a radioimmunoprecipitation assay, with a cut-off value of ≥0.5 nmol/L. The antibody level, combined with mild hypoxemia and decreased forced vital capacity, confirmed myasthenic involvement with incipient respiratory impairment.

Test	Result	Reference Value	Interpretation
Anti-acetylcholine receptor antibodies	Positive (3.2 nmol/L)	<0.5 nmol/L	Consistent with myasthenia gravis
Anti-muscle-specific kinase	Negative	Negative	Not suggestive of the muscle-specific kinase subtype
Electromyography (repetitive nerve stimulation)	No significant decrement	NA	Possible false negative
Arterial blood gas (on admission)	pO₂: 67 mmHg, pCO₂: 45 mmHg, pH: 7.37	pO₂: 75–100 mmHg	Mild hypoxemia
Forced vital capacity	1.7 L (↓65% of predicted)	>80% of predicted	Ventilatory compromise

Given the confirmed diagnosis and the presence of incipient ventilatory compromise, treatment was initiated with five days of intravenous human immunoglobulin (IVIG), adjusted to actual body weight at 0.4 g/kg/day (~30 g/day), and oral pyridostigmine was introduced once swallowing capacity had improved. No adverse events related to IVIG administration (e.g., thromboembolic, renal, or infusion reactions) were observed during hospitalization. The administered treatment is summarized in Table 2.

**Table 2:** Therapeutic scheme administered during hospitalization. A summary of pharmacological treatment and ventilatory support provided during the management of a myasthenic crisis. Intravenous immunoglobulin was administered for five days alongside oral pyridostigmine, resulting in a positive clinical response. Supplemental oxygen was discontinued after respiratory stabilization.

Medication	Dose	Route	Frequency	Duration	Indication
Intravenous immunoglobulin	0.4 g/kg/day (~30 g/day)	Intravenous	Once daily	5 days	Myasthenic crisis with respiratory compromise
Pyridostigmine	60 mg	Oral	Every 6 hours	Continued upon discharge	Improvement of weakness and dysphagia
Supplemental oxygen	2 L/minute	Nasal cannula	Continuous	48 hours	Hypoxemia and resting dyspnea

The patient showed progressive respiratory improvement from the fifth day, enabling the withdrawal of supplemental oxygen. He was discharged on the seventh day of hospitalization, walking independently, and was referred for ambulatory follow-up by the neurology department. As the diagnosis corresponded to generalized MG, a chronic treatment plan was established, consisting of continued oral pyridostigmine and outpatient clinical surveillance. Given the absence of thymoma and stable clinical evolution, the introduction of corticosteroids or immunosuppressive agents was deferred, pending reassessment at follow-up.

## Discussion

HTLV-1 primarily infects CD4+ T lymphocytes, but can also be found in CD8+ T cells, as well as in monocytes and dendritic cells. The virus’s genome encodes structural proteins (Gag, Pol, and Env) and regulatory proteins such as Tax and HBZ. These viral products have been associated with both malignant transformation and immune dysfunction [8].

The Tax protein promotes cellular proliferation and has been associated with lymphoproliferative diseases. HBZ suppresses viral promoter activity, allowing viral persistence in infected cells and contributing to chronic infection [2,8]. Experimental studies have shown that thymic epithelial cells express HTLV-1 receptors and can be infected both by free viral particles and via cell-to-cell contact. This infection induces expression of anti-apoptotic genes, such as *BCL-2*, as well as chemokines. Although our text initially referenced CCL2, which primarily recruits monocytes, more relevant evidence points to CXCL10 as the key chemokine mediating the migration of infected T lymphocytes to the central nervous system [9].

A thymic infection may profoundly disturb immune equilibrium. Exacerbated activation of CD4+ T cells producing IFN-γ, IL-17A, and IL-21 has been reported, creating a proinflammatory thymic environment. Expansion of double-positive CD4+/CD8+ lymphocytes with high inflammatory and neurotoxic capacity has also been described [10,11].

Among the most critical immunological alterations is the dysfunction of Tregs. A reduction in the number and suppressive capacity of Tregs in HTLV-1 infection is well documented and is linked to unstable expression of the FOXP3 transcription factor, in part due to aberrant HBZ expression. Tax further impairs the TGF-β signalling pathway, thereby reducing immunomodulatory tone [12]. While no cellular immunophenotyping or flow cytometry was performed for our patient, these molecular pathways are relevant for contextualizing the potential mechanism of autoimmunity in this case.

In this immunological context, MG can be interpreted as a clinical consequence of central tolerance failure. MG is characterized by autoantibodies targeting the AChR, MuSK, or LRP4 [13]. The thymus is not only central to T-cell maturation but is also the origin of anti-AChR autoantibodies. In MG patients with thymic hyperplasia or thymoma, Treg dysfunction allows autoreactive T clones to interact with B cells in germinal centers, fostering autoantibody production. This process may also occur in the absence of thymic structural abnormalities [13].

Although the exact pathogenic mechanism connecting HTLV-1 infection with MG remains under investigation, several plausible pathways have been proposed. These include: (1) chronic thymic infection and Treg depletion; (2) the generation of molecular mimicry between viral and neuromuscular junction antigens; and (3) bystander activation of autoreactive lymphocytes in a proinflammatory milieu. In vitro studies have shown that HTLV-1-infected epithelial cells can transfer viral particles to CD4+ T cells, perpetuating immune activation [9].

In the present case, the coexistence of HAM/TSP and MG in the absence of thymoma suggests that chronic HTLV-1 infection may have altered the thymic immune environment sufficiently to trigger or unmask latent autoimmunity. While no definitive causal inference can be made, this case adds to the limited number of published reports exploring this intersection [14].

Although the association between HTLV-1 and MG is rarely reported, the growing number of anecdotal cases, combined with the known immunomodulatory effects of HTLV-1, supports the hypothesis that persistent retroviral infection may contribute to autoimmune dysregulation under certain conditions [3].

In Mexico, where this case was documented, the seroprevalence of HTLV-1 is estimated to range from 0.05% to 0.2%, based on regional studies of blood donors [15]. However, the incidence of HAM/TSP remains largely underreported at the national level, likely due to limited surveillance and clinical recognition. In contrast, the prevalence of myasthenia gravis is estimated at 11-14 cases per 100,000 inhabitants in Mexico, according to national epidemiological data [16]. Based on these figures, the probability of coincidental co-occurrence in a single individual is exceedingly low, well below one per million, suggesting that this association may reflect a genuine pathophysiological link rather than a mere coincidence.

## Conclusions

Although the direct association between HTLV-1 infection and MG is not widely recognized in the clinical literature, several experimental studies have demonstrated that this retrovirus can profoundly alter the microenvironment of the thymus. The infection of thymic epithelial cells and the dysfunction of Tregs, induced by the expression of viral proteins such as HBZ and Tax, generate an environment that is both proinflammatory and inefficiently tolerogenic. This disruption favors the activation and persistence of autoreactive CD4+ T-cell clones that can induce the production of autoantibodies by B lymphocytes, including those directed against the AChR. Leakage of these autoantibodies into the bloodstream is the main pathophysiological mechanism in MG. This clinical case, in which a patient with a history of tropical spastic paraparesis due to HTLV-1 developed an immunologically confirmed myasthenic crisis, suggests a potential link between the two conditions via thymic autoimmune mechanisms. While it is not possible to establish a direct causal relationship, the coexistence of these conditions supports the hypothesis that HTLV-1 may trigger or modulate MG pathogenesis. Further clinical and translational research is needed to clarify this association, identify risk markers, and inform immunological surveillance strategies in patients with chronic HTLV-1 infection. Specific approaches could include the prospective screening of HTLV-1 carriers for anti-AChR antibodies, as well as the establishment of longitudinal cohorts incorporating thymic imaging and single-cell sequencing, to improve our understanding of the dynamics of thymic immune dysfunction in this context.
